# From Mouth to Brain: Neuroendocrine Markers Play as a Crosstalk Among Oral and Neurodegenerative Diseases

**DOI:** 10.3389/fendo.2019.00378

**Published:** 2019-06-12

**Authors:** Marco Tatullo, Bruna Codispoti, Irina Makeeva, Caterina Benincasa, Gianrico Spagnuolo

**Affiliations:** ^1^Biomedical Section, Tecnologica Research Institute, Crotone, Italy; ^2^Department of Therapeutic Dentistry, Sechenov University Russia, Moscow, Russia; ^3^Department of Neurosciences, Reproductive and Odontostomatological Sciences, Università di Napoli Federico II, Naples, Italy

**Keywords:** oral cancer, neuroendocrine cancer, predictive markers, predictive medicine, early diagnosis

## Abstract

The neuroendocrine system consists of various cells distributed in non-endocrine functional structures, able to synthesize amines and peptides with both local (paracrine) and systemic (endocrine) effects. The presence of such cells, belonging to the neuroendocrine system, is highlighted by the presence of neuroendocrine markers: the most suggestive are chromogranin A, synaptophysin, S-100B protein and glial fibrillary acidic protein. The presence of neuroendocrine markers is commonly associated to the occurrence of neuroendocrine cancers, currently representing the 0.5 percent of all malignant tumors. Nevertheless, neuroendocrine markers have been found to be overexpressed in rare oral neuroendocrine tumors, but also in quite common inflammatory conditions, such as severe periodontitis. The monitoring of neuroendocrine markers is, thus, a common factor of interest among dentistry and neurology: the analysis of neuroendocrine markers in oral diseases may be predictive and prognostic about the severity of neurological diseases, such as lateral amyotrophic sclerosis and traumatic brain injuries. The aim of this mini-review is to highlight the role of neuroendocrine molecules as advantageous diagnostic and prognostic markers for both oral diseases and neurodegenerative disorders.

## Introduction

In the last 10 years, a growing interest about the molecular biology of cancer has allowed to identify several tumor-related biomarkers. Such markers are typically described as: prognostic markers and predictive markers. The presence/absence of a specific marker is useful for choosing the proper treatment for oncological patients, however, the right therapy is not synonym of a response to treatment. Predictive markers, conversely, have the objective of evaluating the ability of a specific treatment to improve the clinical response to oncological therapies (1).

Nowadays, predictive markers have an increasingly important function in evaluating new therapies, and in understanding how drugs can interfere with some biological mechanisms. In the field of oncology, these biomarkers are generally molecules involved in the development of neoplasia; usually, these molecules are produced directly by the tumor, and they can be enzymes or hormones that correlate with the growth of cancer cells, or substances produced by the body, in response to the oncological growth (2). Predictive markers for oral tumors are located in several tissues and they are involved in several pathogenesis: their knowledge can be useful to manage the proper therapeutic approach (3).

Recently, immunohistochemical detection of S-100B protein, Glial Fibrillary Acidic Protein (GFAP), synaptophysin or chromogranin-A (CgA) have been studied in oral tissues, such as bundles of human dental pulp; this allowed to find potential different locations of such markers, depending from the presence of physiological or inflamed pulp; these markers have also been investigated about their use for the determination of local inflammation (4) and rare forms of oral cancer (5). These molecules are also overexpressed following brain trauma and neurodegenerative disease (Table 1).

**Table 1:** Main features of neuroendocrine markers in oral and neurological diseases.

**Markers**	**Mass**	**Location**	**Related info**	**Neuroendocrine markers in oral disease**	**Neuroendocrine markers in oral cancer**	**Neuroendocrine markers in neurological disease**
Chromogranin A	49-kDa	Neuroendocrine tissues	CgA is a constitutive secretory product of most neuroendocrine tumors.	CgA is produced by human submandibular gland HSG and secreted into saliva and is present into inflamed pulp and in caries with concomitant mild periodontitis.	Immunoreactivity studies have confirmed the presence of Chromogranin A in high-grade neuroendocrine tumors (HGNEC)	CgA has pathological features in Amyotrophic lateral sclerosis. CgA was shown to interact in a chaperone-like manner with ALS mutant SOD1 and mediate its secretion CgA is reflective of disease severity and the affective state of ALS patients.
Synaptophysin	38-kDa	Synaptic vesicles of neurons	Ultra-structurally, synaptophysin is present in micro-vesicles, whereas chromogranin is present in secretory granules.	Synaptophysin is present into inflamed pulp and sub-odontogenic layer of the dental pulp.	Immuno-histochemistry typically detected the presence of Chromogranin in the tumor of the tongue	Synaptophysin is related to Alzheimer disease: synaptic abnormalities in the hippocampus correlate with the severity of neuropathology and memory deficit in individuals with Alzheimer disease.
S-100B protein	21-kDa	Astrocytes, certain neuronal populations, Schwann cells	S100B is released in the extracellular space in response to glutamate, serotonin, TNF, IL-1β, beta-amyloid peptides and lysophosphatidic acid.	S-100B protein is present into inflamed pulp.		S100B is a useful neurobiochemical marker of brain damage such as in circulatory arrest, stroke, traumatic brain injury and Alzheimer disease.
Glial Fibrillary Acidic Protein (GFAP)	50-kDa	Astrocytes and ependymal cells of the central nervous system.	The gold biomarker of all types of nervous system injuries	Glial Fibrillary Acidic Protein (GFAP) is present into inflamed pulp.		GFAP is a biomarker for traumatic brain injuries (TBI): this biomarker may predict the severity of injury.

### Chromogranin A

Chromogranin A is a glycoprotein located in the neuroendocrine system; it is abundantly secreted by neurons and endocrine cells together with other hormones and neuropeptides. CgA is involved in several physiological functions, such as calcium regulation, vasoconstriction, metabolism of glucose and bacterial defense, and CgA represents the main central modulator of the neuroendocrine system. Chromogranin A can be produced in specific stress-related physiological conditions (6). CgA is a constitutive secretory product of most neuroendocrine tumors. Recent studies have assessed that patients suffering from neuroendocrine diseases, especially oncological pathologies, showed increased plasma level of Chromogranin A, although some drugs as well as some overlapped organ insufficiency were able to alter such values. Thus, Chromogranin A can be considered as a reliable diagnostic and prognostic marker, after a careful evaluation of patients' general conditions (7).

Chromogranin A is the main secreted protein of neuroendocrine (NE) cells and is produced at high levels by those cell types. Pulmonary neuroendocrine tumors express large amounts of NE markers including synaptophysin, chromogranin A, and N-CAM (also known as CD56). These proteins are typically produced by NE tumors, with diverse levels in the different neoplastic classes, indeed elevated frequencies are detected in carcinoids and in atypical carcinoids respect than in large cell and in small cell neuroendocrine carcinomas. Furthermore, elevated expression of pancreatic polypeptide, α and β subunits of human chorionic gonadotropin and chromogranin A are present in all types of carcinoid neoplasms (8).

In neuroendocrine gastro-intestinal cancers the plasmatic levels of chromogranin A are augmented in more than 80% of affected patients thus, is considered the main marker for this tumor type.

Chromogranin A also exert a prognostic value, especially in general midgut carcinoids cases, because of the existing correlation with the tumor load.

The presence of elevated levels of CgA is detectable before radiographic evidence of gastro intestinal NE cancers occurrence (9).

### Synaptophysin

Synaptophysin is a 38 kD glycoprotein which represents the principal cytoplasmic calcium-binding protein of the membrane of synaptic vesicles of neurons. Synaptophysin and chromogranin could be considered as complementary, common NE markers; indeed, alike to CgA, synaptophysin is expressed by NE cells and consequently in their corresponding tumors.

However, although secretory granules host chromogranin, synaptophysin is mainly located into micro-vesicles.

Synaptophysin is also expressed by adrenal cortical tissues and in related tumors, for this reason it could not be considered as an exclusive marker for NE cells (10).

Furthermore, immunoreactivity for synaptophysin alone is not sufficient for the definitive classification of a neoplasm as neuroendocrine; nevertheless, its presence contributes to recognize neuroendocrine features of a tissue. Synaptophysin is widely expressed in large cell undifferentiated neuroendocrine carcinoma. In a clinical study, small cell lung carcinomas stained positive for synaptophysin in the 79% of patient, whereas 47 to 60% of cases were positive for chromogranin staining. Moreover, 8% of non-small cell carcinomas showed synaptophysin labeling (11).

### S-100B Protein

S-100B protein is a 21kDa acidic protein broadly expressed in astrocytes, Schwann cells, myeloid-derived cells, and in definite populations of neurons. The secretion of S-100B in the extracellular space is activated by serotonin, glutamate, beta-amyloid peptides, TNF, lysophosphatidic acid, and IL-1β. After secretion, S-100B is able to produce their paracrine and autocrine effects on neuronal cells and glia (12).

### Glial Fibrillary Acidic Protein (GFAP)

Glial fibrillary acidic protein (GFAP) represents a structural protein of the intermediate filaments integral to cytoskeleton. This protein is responsible for the morphology of astrocytes and it also regulates the reactive responses of astrocytes during aging. In an *in vivo* study, O'Callghan et al. demonstrate, by histopathological analyses, the increased expression of GFAP in “all types of nervous system injuries” (13).

## Neuroendocrine Markers in Oral Diseases

Human dental pulp expresses neuron-specific enolase (NSE) and S-100B protein but showed negative immunolabeling for chromogranin A and peripherin (PRP). This expression pattern is present both in normal conditions and during pulp inflammation with an amplified level of S100B and NSE of the nerve fibers detected in inflamed pulp compared to healthy samples. These evidences propose the potential role of S100B and NSE as markers for dental innervation. Moreover, these proteins may be applied for determination of the amplitude of inflamed area and could be predictive of the evolution of the inflammation process in pulp therapy. The lack of a positive chromogranin A immunostaining in human dental pulp indicate the probable absence of these common neuroendocrine protein, while the role of PRP in pulp tissues need further investigations (14).

Neuroendocrine markers are located in specific cytotypes known as NE cells, characterized by asymmetrical shape and large dimensions and by the presence of cytoplasmic granules and round nucleus. The presence of NE cells in the dental pulp still need to be clarified. In another study, the immunohistochemical analysis of human dental pulp with streptavidin-biotin labeling revealed the expression of NSE, CgA, S-100B, GFAP, and synaptophysin all common neural crest related markers. The ontogenesis of dental pulp from neural crest is commonly accepted, developing teeth (15) contain cells deriving from neural crest, responsible for dental pulp innervation, this cytotype secreted nerve growth factors, glial and cerebral neurotrophic factor, and control the development of dopaminergic fibers into the pulp during tooth growth (16).

The presence of synaptophysin was also registered in the para-odontoblastic zone of dental pulp.

Ulterior immunohistochemical studies with Ab against NEC antigens (CgA, synaptophysin, and NSE) identified rare positive cells only in the sub-odontogenic layer of the pulp. Immunopositive cells for NSE and synaptophysin were found to be located nearly to the nerve fibers, but CgA was not detected in nerve fibers (17).

Immunopositive NE cells were detected in caries, the number of positive cells concomitantly increased with the rising gravity of inflammation with a maximum expression corresponding to periodontitis. The overwhelming majority of cells were detected by antibodies to chromogranin A. It could be supposed that NE cells located at the interface between pulp and dentin produce peptides that induce local (paracrine) and distant (endocrine) effects, that influences the behavior of other cell types. Indeed, NE cells are involved in the regulation of the microcirculation, of the immune response and in the proliferation of fibroblasts. The detected augmented occurrence of nerve fibers in caries and pulpitis can be related to their participation in the regulation of the process of inflammation. Poor reactivity of NE cells into normal pulp to nerve crest markers can be explained by the declining expression of these antigens during cytodifferentiation. According to the described results, this ability is induced under pathological states (16).

The correlation between the salivary glands and the nervous system underlines the important role of secreted proteins as important biomarkers for nervous system behavior. The presence and localization of CgA in the human submandibular gland using various methods has been investigated. CgA was copiously localized in serous and ductal cells of the submandibular gland, as revealed by immunohistochemistry and *in situ*-hybridization. Immunopositive reaction was stronger in serous cells than in ductal cells. In addition, a significant immunoreactivity for CgA was observed in the saliva matrix of ductal cavities, as demonstrated by immunoelectron microscopy and western blotting. The obtained resulted supported the hypothesis that CgA is produced by human submandibular gland and secreted into saliva (18).

In a case-control study involving 30 subjects affected by chronic periodontitis and a control group comprising of 30 healthy patients, the levels of CgA in plasma saliva were determined by ELISA quantification. A significantly higher CgA amount was found in plasma salivary samples of individuals affected by chronic periodontitis respect to control patients (19).

## Neuroendocrine Markers in Oral Cancers

Neuroendocrine neoplasms commonly occur in the gastrointestinal tract and in lung (20).

In the head and neck district, the majority of neuroendocrine tumor arise in the larynx, and in the salivary glands (21); while the intra-oral mucosa represents the rarest primary sites where neuroendocrine malignancies have been described.

Only few cases of neuroendocrine carcinomas (NECs) have been reported in the oral cavity.

Neuroendocrine carcinoma of the tongue involves alteration on neuroendocrine markers expression. In a clinical study, the analysis of tongue biopsy revealed the presence of a squamous mucosa with neoplasm and identified group of cells characterized by round nuclei and light cytosol. Immunohistochemical study evidenced a positive staining for cytokeratin, CgA, NSE, and synaptophysin and negative reaction for leukocyte common antigen (22).

The most common type of pulmonary neoplasm, small cell neuroendocrine carcinoma (SNEC) is a very aggressive malignancy with a high tendency for metastatic dissemination. Extrapulmonary SNECs characterize the 2.5–5% of all SNECs (23), and only the 10–16% of which involve head and neck localization.

The general prognosis of head and neck SNEC is poor because of the propensity for aggressive local invasion and a strong tendency for distant metastasis formation (24).

In a 73-year-old male a rare case of SNEC of the gingiva has been registered. The diagnosis of SNEC was established on morphology and validation of origin by specific stains (25). SNEC malignancies are generally composed of group of rounds to spindle-shaped cells with dense nuclei and scarce cytoplasm. Moreover, mitotic activity and cell necrosis are analyzed.

Definitive confirmation of SNEC tumor cells presence has been established by immunoreactivity for cytokeratin and neuroendocrine markers such as chromogranin A, synaptophysin, neuron-specific enolase, and CD56 (26).

A case of high-grade neuroendocrine tumors was registered in a 75-year-old man on the left lateral anterior tongue. The subject showed a 5 mm diameter ulcerated wound on the lateral border of the tongue, immunohistochemical analyses of biopsy revealed the typical features of a poorly differentiated neuroendocrine carcinoma of small cell type. The tumor cells were strongly positive for neuroendocrine markers including chromogranin A, synaptophysin and CD56 (27).

## Neuroendocrine Markers in Neurological Diseases

Neuroendocrine proteins including CgA, S-100B protein, synaptophysin, GFAP protein, and neurofilaments, typically located in neural tissues, are commonly used as markers for neuroendocrine carcinomas. Their expression could be altered in neurogenic disorders.

Several studies connected CgA to pathological features of Amyotrophic lateral sclerosis (ALS). For instance, CgA was shown to interact in a chaperone-like manner with ALS mutant SOD1 and mediate its secretion, there was a significant loss of CgA expressing neurons accompanied by decreased CgA density in the neuropil and an accumulation of CgA in the remaining neurons (28).

Toda et al. performed a comparative study evaluating the levels of CgA in saliva of patients suffering of vascular dementia, moderate ALS and terminal ALS. They found increased values of salivary CgA only in patients affected by terminal ALS. Furthermore, the concentration of CgA is related to the Escorial score of emotional functioning rather than that of corporeal mobility, language and nutrition. The obtained results confirmed that CgA expression correlate with ALS severity and with the affective conditions of patients (29).

A pivotal hypothesis was that hippocampal synaptic defects of patients affected by Alzheimer disease (AD) were connected with the severity of cognitive impairment.

Synaptophysin levels were quantified by immunoblotting of synaptic membrane fractions isolated from entorhinal cortex, hippocampus, occipital cortex, and caudate nucleus. The concentration of synaptophysin were decreased in hippocampus when comparing AD patients to controls. These data allow to conclude that synaptic aberrations in the hippocampus are connected with the severity of memory deficit and neuropathology in individuals with AD (30).

S-100B could be considered a biomarker of brain damage in stroke, circulatory arrest and traumatic brain injury. S-100B is also associated with chronic neurological illness and neurodegenerative diseases like Alzheimer. Additionally, S-100B may be predictive of prognosis and of the efficiency of treatment (31).

GFAP resulted up-regulated in central nervous system damage following the reactive gliosis that typically occur after traumatic brain injuries. Numerous studies demonstrate increasing levels of GFAP in plasma of patients subjected to traumatic brain injuries, thus the quantification of this biomarker may be predictive of the severity of damage (32).

## Conclusions

Current biomarkers are molecules aimed to allow predictable and targeted therapy, customized on the specific case, even if several markers can be related to different seats of activity. For example, the evaluation of Epidermal Growth Factor Receptor (EGFR) expression is highly necessary for therapeutic planning of anti-EGFR drugs in patients with lung or oral cancer (33).

**Figure 1:**
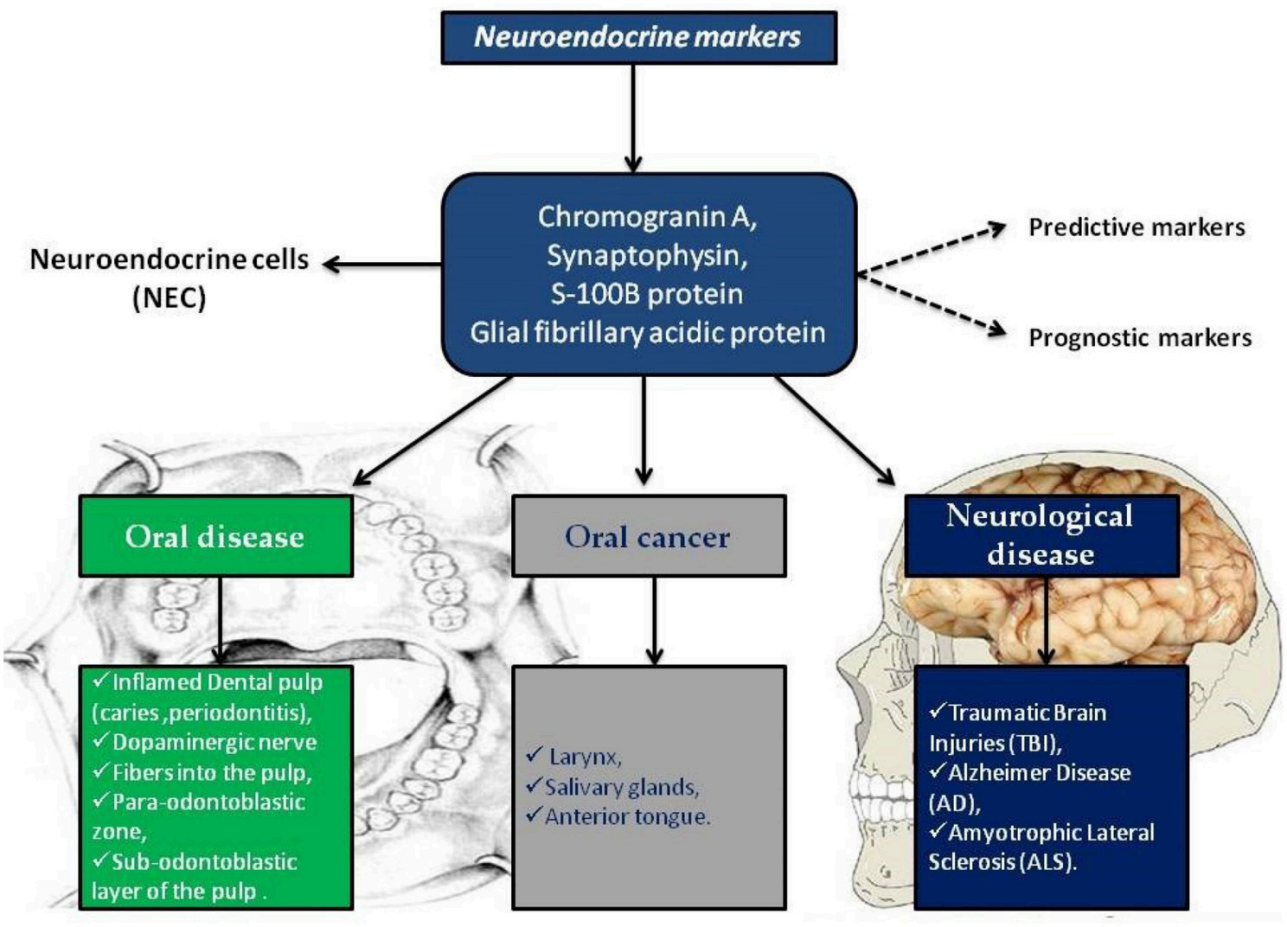
Neuroendocrine markers distribution in oral disease, in oral cancer and in neurological diseases.

In this scenario, the identification of predictive markers becomes a significant diagnostic and prognostic factor, other than, it would favor the use of certain drugs in a specific patient population. Chromogranin A, synaptophysin, S-100B protein and GFAP are well known neuroendocrine markers involved in cancer and in neurological diseases. In the brain their expression is used as predictable of severity of neuro-pathologies including ALS, AD, and traumatic brain injuries. Interestingly, neuroendocrine markers are also present in oral tissues such as dental pulp and salivary glands, probably due to the derivation from neural crest of oral structures (Figure 1).

The take-home message is to improve the investigation on neuroendocrine markers, as they are an important cluster of molecules useful for the identification and characterization of rare forms of cancer, such as NE oral cancers, and they can be also considered valuable biomarkers in oral inflammatory diseases, such as periodontitis. Furthermore, the altered expression of neuroendocrine molecules in neurodegenerative disease would confirm their application also in the diagnosis of neurological pathologies. In conclusion, the analysis of NE molecules represents a valuable tool to be better considered in the future clinical applications.

## Data Availability

No datasets were generated or analyzed for this study.

## Author Contributions

The literature search was independently performed by 3 authors (MT, BC, and CB). The manuscript was drafted by all the authors (MT, BC, IM, CB, and GS).

### Conflict of Interest Statement

The authors declare that the research was conducted in the absence of any commercial or financial relationships that could be construed as a potential conflict of interest. The handling Editor declared a past collaboration with one of the authors, MT.
